# Tetra­kis(μ-4-ethyl­benzoato-κ^2^ *O*:*O*′)­bis­[(4-ethyl­benzoic acid-κ*O*)copper(II)]

**DOI:** 10.1107/S1600536808015924

**Published:** 2008-06-19

**Authors:** Abraham C. Sunil, Barend C. B. Bezuidenhoudt, J. Marthinus Janse van Rensburg

**Affiliations:** a Department of Chemistry, University of the Free State, PO Box 339, Bloemfontein 9300, South Africa

## Abstract

The molecule of the title compound, [Cu_2_(C_9_H_9_O_2_)_4_(C_9_H_10_O_2_)_2_], lies on a center of inversion. It consists of four bridging ethyl­benzoate ligands, forming a cage around two Cu atoms in a *syn*–*syn* configuration, and two monodentate ethyl­benzoic acid ligands bonded apically to the square-planar Cu atoms. The Cu⋯Cu distance is 2.6047 (5) Å.

## Related literature

For the synthesis of aromatic carboxylic acids, see: Kaeding (1967 ▶). For tetra­kis(μ_2_-2-methyl­benzoato)bis­(2-methyl­benzoic acid)dicopper(II), see: Sunil *et al.* (2008 ▶). For tetra­kis(μ_2_-2-fluoro­benzoato)bis­(2-fluoro­benzoic acid)dicopper(II), see: Valach *et al.* (2000 ▶). For tetra­kis(μ_2_benzoato) bis­(2-fluoro­benzoic acid)dicopper(II), see: Kawata *et al.* (1992 ▶). For tetra­kis-[μ-(2-phenoxy­benzoato-*O*,*O*′)]bis­[(2-phenoxy­benzoic acid)copper(II)], see: Mak & Yip (1990 ▶).
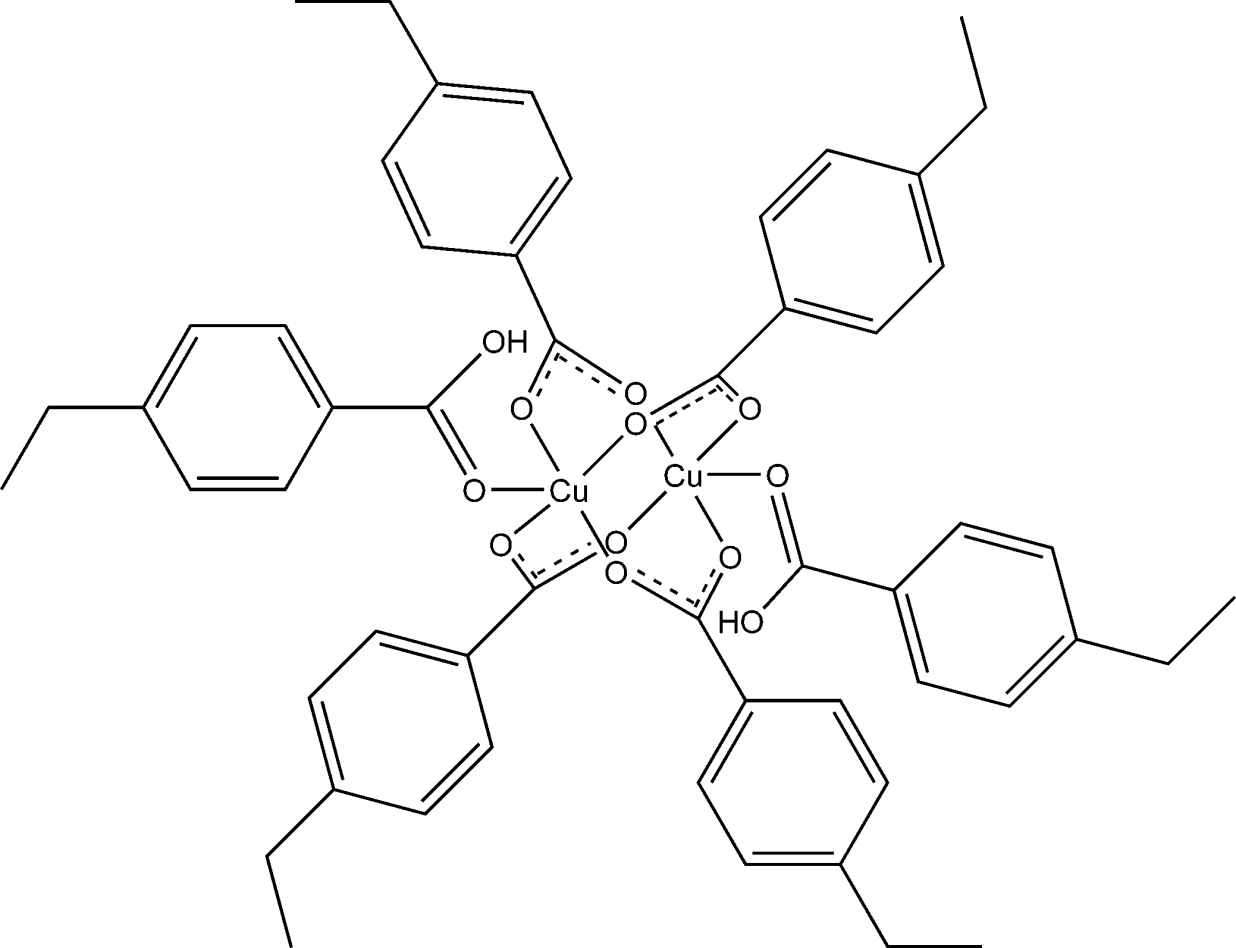


## Experimental

### 

#### Crystal data

[Cu_2_(C_9_H_9_O_2_)_4_(C_9_H_10_O_2_)_2_]
*M* *_r_* = 1024.07
Triclinic, 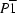

*a* = 10.6167 (5) Å
*b* = 10.7394 (7) Å
*c* = 10.8096 (7) Å
α = 81.848 (3)°
β = 88.594 (3)°
γ = 79.468 (2)°
*V* = 1199.47 (12) Å^3^
*Z* = 1
Mo *K*α radiation
μ = 0.95 mm^−1^
*T* = 100 (2) K
0.54 × 0.4 × 0.39 mm

#### Data collection

Bruker Kappa APEXII diffractometer
Absorption correction: multi-scan (*SADABS*; Bruker, 2004 ▶) *T*_min_ = 0.628, *T*_max_ = 0.708
15971 measured reflections
5683 independent reflections
4721 reflections with *I* > 2σ(*I*)
*R*_int_ = 0.040

#### Refinement

*R*[*F* ^2^ > 2σ(*F* ^2^)] = 0.040
*wR*(*F* ^2^) = 0.103
*S* = 1.03
5683 reflections
311 parameters
H-atom parameters constrained
Δρ_max_ = 0.50 e Å^−3^
Δρ_min_ = −0.37 e Å^−3^

### 

Data collection: *APEX2* (Bruker, 2005 ▶); cell refinement: *SAINT-Plus* (Bruker, 2004 ▶); data reduction: *SAINT-Plus* and *XPREP* (Bruker, 2004 ▶); program(s) used to solve structure: *SIR97* (Altomare *et al.*, 1999 ▶); program(s) used to refine structure: *SHELXL97* (Sheldrick, 2008 ▶); molecular graphics: *DIAMOND* (Brandenburg & Putz, 2005 ▶); software used to prepare material for publication: *WinGX* (Farrugia, 1999 ▶).

## Supplementary Material

Crystal structure: contains datablocks global, I. DOI: 10.1107/S1600536808015924/fi2063sup1.cif

Structure factors: contains datablocks I. DOI: 10.1107/S1600536808015924/fi2063Isup2.hkl

Additional supplementary materials:  crystallographic information; 3D view; checkCIF report

## Figures and Tables

**Table 1:** Selected bond lengths (Å)

Cu1—O3	1.9498 (15)
Cu1—O4	1.9501 (16)
Cu1—O2	1.9593 (16)
Cu1—O1	2.0040 (16)
Cu1—O5	2.1761 (15)
Cu1—Cu1^i^	2.6047 (5)

Symmetry code: (i) 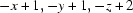.

**Fig. 1.**
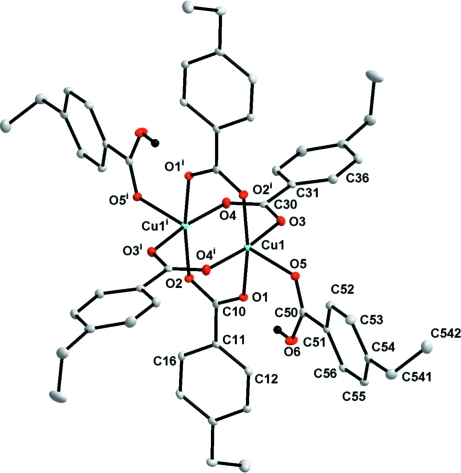
A view of (I) showing the atom-numbering scheme with displacement ellipsoids at the 30% probability level, non labelled atoms are symmetric equivalents. For the phenyl C-atoms, the first digit indicates ring number and the second digit the position of the atom in the ring. Symmetry code: 1 - x, 1 - y, 2 - z.

**Fig. 2.**
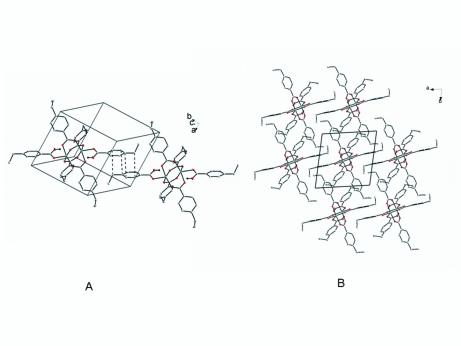
(A) Hacked lines indicate overlap between ethylbenzoic groups of neighbouring molecules. (B) Indication of pseudo-hexagonal close packing along the c axis.

## supplementary crystallographic information

### Comment

The title compound forms part of the copper(II) complexes of the type [Cu_2_(RCO_2_)_4_*L*_2_] (*R*=aryl, *L*=monodentate ligand). This type of complex forms tetra-(carboxylato-O,*O*') bridges and four of the carboxylate groups hold together two Cu atoms (Fig. 1). The Cu···Cu distance in the title compound is 2.6047 (5) Å, probably displaying weak orbital interaction considering that the van der Waals radius of copper is 2.32 Å. The axial sites of each copper atom are bonded to a monodentate *p*-ethylbenzoic acid ligand. In turn the acid protons are hydrogen bonded to the cage carboxylate O atoms, O—H···O = 166.79° and O···O = 2.645 Å.

Neighbouring molecules stack with overlap between the axially bonded phenyl rings displaying a centroid to centroid distance of 4.2918 (3) Å and an interplanar distance of 3.6277 Å (Fig. 2 A). This inter-molecular interaction influence the dihedral angle displayed between the phenyl rings from the axially bonded monodentate ligands and the carboxylic oxygen plane, O1, O2, O1^i^ and O2^i^ (i = 1 - *x*, 1 - *y*, 2 - *z*). Molecular packing in the (0 0 h) plane is in a puckered pseudo-hexagonal close packing fashion. This close packing is stabilized by soft inter-molecular C···H contacts ranging from 2.720–2.813 Å (Fig. 2B).

### Experimental

The complex [Cu_2_(C_9_H_10_O_2_)_4_(C_9_H_11_O_2_)_2_] was prepared by heating 4-ethylbenzoic acid (1.77 g, 11.81 mmol), copper carbonate (0.74 g, 3.34 mmol) and magnesium oxide (0.20 g, 4.98 mmol) under reflux, in toluene (15 ml) for 60 h. The product was extacted and crystallized from diethyl ether to yield a blue crystalline solid. (Yield: 80%)

### Refinement

The H atoms were positioned geometrically and refined using a riding model with fixed C—H distances of 0.93 Å (CH) [*U*_iso_(H) = 1.2*U*_eq_] and 0.96 Å (CH_3_) [*U*_iso_(H) = 1.5*U*_eq_] respectively. Initial positions of methyl H-atoms were obtained from Fourier difference maps and refined as a fixed rotor.

The highest density peak is 0.50 located 0.65 Å from C14 and the deepest hole is -0.37 located at 0.68 Å from Cu1.

### Crystal data

**Table:**

[Cu_2_(C_9_H_9_O_2_)_4_(C_9_H_10_O_2_)_2_]	*Z* = 1
*M**_r_* = 1024.07	*F*_000_ = 534
Triclinic, *P*1	*D*_x_ = 1.418 Mg m^−^^3^
Hall symbol: -P 1	Mo *K*α radiation λ = 0.71069 Å
*a* = 10.6167 (5) Å	Cell parameters from 4441 reflections
*b* = 10.7394 (7) Å	θ = 2.5–28.2º
*c* = 10.8096 (7) Å	µ = 0.95 mm^−^^1^
α = 81.848 (3)º	*T* = 100 (2) K
β = 88.594 (3)º	Cuboid, blue
γ = 79.468 (2)º	0.54 × 0.4 × 0.39 mm
*V* = 1199.47 (12) Å^3^	

### Data collection

**Table:**

Bruker Kappa APEXII diffractometer	4721 reflections with *I* > 2σ(*I*)
Monochromator: graphite	*R*_int_ = 0.040
*T* = 100(2) K	θ_max_ = 28º
ω and φ scans	θ_min_ = 2.5º
Absorption correction: multi-scan(SADABS; Bruker, 2004)	*h* = −7→14
*T*_min_ = 0.628, *T*_max_ = 0.708	*k* = −14→14
15971 measured reflections	*l* = −14→14
5683 independent reflections	

### Refinement

**Table:**

Refinement on *F*^2^	H-atom parameters constrained
Least-squares matrix: full	*w* = 1/[σ^2^(*F*_o_^2^) + (0.0447*P*)^2^ + 0.9309*P*] where *P* = (*F*_o_^2^ + 2*F*_c_^2^)/3
*R*[*F*^2^ > 2σ(*F*^2^)] = 0.040	(Δ/σ)_max_ = 0.001
*wR*(*F*^2^) = 0.103	Δρ_max_ = 0.50 e Å^−^^3^
*S* = 1.04	Δρ_min_ = −0.37 e Å^−^^3^
5683 reflections	Extinction correction: none
311 parameters	

### Special details

**Table:**

Experimental. The intensity data was collected on a Bruker X8 Apex II 4 K Kappa CCD diffractometer using an exposure time of 2 s/frame. A total of 1507 frames were collected with a frame width of 0.5° covering up to θ = 28.0° with 98.3% completeness accomplished.
Geometry. All e.s.d.'s (except the e.s.d. in the dihedral angle between two l.s. planes) are estimated using the full covariance matrix. The cell e.s.d.'s are taken into account individually in the estimation of e.s.d.'s in distances, angles and torsion angles; correlations between e.s.d.'s in cell parameters are only used when they are defined by crystal symmetry. An approximate (isotropic) treatment of cell e.s.d.'s is used for estimating e.s.d.'s involving l.s. planes.

### Fractional atomic coordinates and isotropic or equivalent isotropic displacement parameters (Å^2^)

**Table:**

	*x*	*y*	*z*	*U*_iso_*/*U*_eq_	
Cu1	0.45033 (3)	0.46672 (2)	0.90347 (2)	0.01213 (9)	
O6	0.58444 (17)	0.40202 (18)	0.62540 (16)	0.0239 (4)	
H6	0.599	0.4445	0.679	0.036*	
O1	0.60816 (15)	0.51776 (15)	0.82165 (14)	0.0152 (3)	
O3	0.36215 (15)	0.64398 (14)	0.86973 (14)	0.0166 (3)	
O4	0.54987 (16)	0.29874 (15)	0.96412 (14)	0.0181 (3)	
O2	0.30749 (15)	0.42705 (15)	1.01089 (14)	0.0167 (3)	
O5	0.40263 (15)	0.39621 (15)	0.73471 (14)	0.0168 (3)	
C51	0.4404 (2)	0.2808 (2)	0.5616 (2)	0.0144 (4)	
C11	0.8123 (2)	0.58002 (19)	0.80261 (19)	0.0124 (4)	
C30	0.3784 (2)	0.7244 (2)	0.94028 (19)	0.0140 (4)	
C53	0.3163 (2)	0.1251 (2)	0.5207 (2)	0.0192 (5)	
H53	0.2542	0.0759	0.546	0.023*	
C55	0.4715 (2)	0.1870 (2)	0.3717 (2)	0.0172 (5)	
H55	0.5134	0.1806	0.2957	0.021*	
C52	0.3464 (2)	0.2079 (2)	0.5971 (2)	0.0172 (5)	
H52	0.3038	0.215	0.6727	0.021*	
C54	0.3778 (2)	0.1138 (2)	0.4054 (2)	0.0169 (5)	
C36	0.2111 (2)	0.8866 (2)	0.8178 (2)	0.0171 (5)	
H36	0.1956	0.823	0.773	0.02*	
C16	0.9223 (2)	0.5936 (2)	0.8637 (2)	0.0145 (4)	
H16	0.9212	0.5918	0.95	0.017*	
C10	0.6968 (2)	0.55566 (19)	0.87604 (19)	0.0133 (4)	
C31	0.3059 (2)	0.8585 (2)	0.9087 (2)	0.0143 (4)	
C34	0.1628 (2)	1.1064 (2)	0.8569 (2)	0.0184 (5)	
C32	0.3321 (2)	0.9558 (2)	0.9709 (2)	0.0191 (5)	
H32	0.3968	0.9384	1.0306	0.023*	
C56	0.5043 (2)	0.2694 (2)	0.4481 (2)	0.0162 (5)	
H56	0.5682	0.3166	0.424	0.019*	
C12	0.8152 (2)	0.5865 (2)	0.6725 (2)	0.0146 (4)	
H12	0.743	0.5777	0.63	0.018*	
C541	0.3421 (3)	0.0255 (2)	0.3213 (2)	0.0235 (5)	
H54A	0.3627	−0.0618	0.3633	0.028*	
H54B	0.3936	0.0313	0.2461	0.028*	
C141	1.1539 (2)	0.6401 (2)	0.5944 (2)	0.0208 (5)	
H14A	1.1803	0.571	0.5452	0.025*	
H14B	1.2228	0.6391	0.6521	0.025*	
C50	0.4725 (2)	0.3652 (2)	0.6484 (2)	0.0155 (5)	
C13	0.9247 (2)	0.6059 (2)	0.6070 (2)	0.0158 (5)	
H13	0.9246	0.6114	0.5203	0.019*	
C15	1.0327 (2)	0.6096 (2)	0.7979 (2)	0.0164 (5)	
H15	1.1059	0.6153	0.8408	0.02*	
C14	1.0360 (2)	0.6173 (2)	0.6679 (2)	0.0152 (4)	
C642	−0.0475 (3)	1.2359 (3)	0.9030 (3)	0.0385 (7)	
H64A	−0.0923	1.1768	0.8717	0.058*	
H64B	−0.0989	1.32	0.8901	0.058*	
H64C	−0.0311	1.21	0.9907	0.058*	
C641	0.0790 (3)	1.2371 (2)	0.8338 (2)	0.0254 (6)	
H64D	0.0628	1.2612	0.7449	0.03*	
H64E	0.1226	1.2997	0.8623	0.03*	
C33	0.2619 (3)	1.0788 (2)	0.9443 (2)	0.0229 (5)	
H33	0.2812	1.1435	0.9852	0.028*	
C35	0.1394 (2)	1.0083 (2)	0.7935 (2)	0.0186 (5)	
H35	0.0746	1.0253	0.7339	0.022*	
C142	1.1323 (3)	0.7677 (3)	0.5072 (3)	0.0323 (6)	
H14C	1.0651	0.7688	0.449	0.048*	
H14D	1.2099	0.7775	0.4623	0.048*	
H14E	1.1085	0.8367	0.5556	0.048*	
C542	0.2016 (3)	0.0542 (2)	0.2846 (2)	0.0277 (6)	
H54C	0.1498	0.0463	0.3583	0.042*	
H54D	0.1855	−0.0053	0.231	0.042*	
H54E	0.1807	0.1397	0.2412	0.042*	

### Atomic displacement parameters (Å^2^)

**Table:**

	*U*^11^	*U*^22^	*U*^33^	*U*^12^	*U*^13^	*U*^23^
Cu1	0.01076 (16)	0.01499 (14)	0.01135 (13)	−0.00383 (10)	0.00010 (10)	−0.00231 (9)
O6	0.0189 (10)	0.0369 (10)	0.0225 (9)	−0.0156 (8)	0.0057 (7)	−0.0139 (7)
O1	0.0110 (8)	0.0221 (8)	0.0148 (7)	−0.0076 (7)	0.0005 (6)	−0.0041 (6)
O3	0.0161 (9)	0.0154 (7)	0.0184 (8)	−0.0022 (6)	−0.0021 (7)	−0.0032 (6)
O4	0.0212 (9)	0.0164 (8)	0.0168 (8)	−0.0025 (7)	−0.0044 (7)	−0.0032 (6)
O2	0.0130 (8)	0.0256 (8)	0.0138 (7)	−0.0087 (7)	0.0014 (6)	−0.0036 (6)
O5	0.0144 (9)	0.0232 (8)	0.0142 (7)	−0.0042 (7)	0.0001 (6)	−0.0059 (6)
C51	0.0119 (12)	0.0176 (10)	0.0129 (10)	−0.0019 (9)	−0.0023 (8)	0.0001 (8)
C11	0.0097 (11)	0.0118 (9)	0.0152 (10)	−0.0021 (8)	−0.0009 (8)	−0.0005 (8)
C30	0.0114 (11)	0.0179 (10)	0.0130 (10)	−0.0047 (9)	0.0049 (8)	−0.0008 (8)
C53	0.0187 (13)	0.0212 (11)	0.0195 (11)	−0.0096 (10)	0.0022 (10)	−0.0010 (9)
C55	0.0149 (12)	0.0217 (11)	0.0139 (10)	0.0000 (9)	0.0025 (9)	−0.0030 (9)
C52	0.0159 (12)	0.0235 (11)	0.0126 (10)	−0.0057 (10)	0.0022 (9)	−0.0015 (9)
C54	0.0152 (12)	0.0178 (11)	0.0169 (11)	0.0007 (9)	−0.0032 (9)	−0.0041 (9)
C36	0.0165 (12)	0.0166 (10)	0.0184 (11)	−0.0035 (9)	0.0004 (9)	−0.0026 (8)
C16	0.0140 (12)	0.0171 (10)	0.0128 (10)	−0.0039 (9)	−0.0025 (9)	−0.0019 (8)
C10	0.0147 (12)	0.0124 (10)	0.0122 (9)	−0.0027 (9)	−0.0021 (8)	0.0009 (8)
C31	0.0111 (11)	0.0164 (10)	0.0155 (10)	−0.0038 (9)	0.0037 (9)	−0.0014 (8)
C34	0.0192 (13)	0.0159 (11)	0.0191 (11)	−0.0037 (9)	0.0064 (9)	0.0001 (9)
C32	0.0186 (13)	0.0215 (11)	0.0174 (11)	−0.0029 (10)	−0.0034 (9)	−0.0040 (9)
C56	0.0107 (12)	0.0200 (11)	0.0180 (11)	−0.0046 (9)	0.0013 (9)	−0.0015 (9)
C12	0.0112 (12)	0.0175 (10)	0.0161 (10)	−0.0041 (9)	−0.0022 (9)	−0.0029 (8)
C541	0.0256 (14)	0.0240 (12)	0.0239 (12)	−0.0073 (11)	0.0018 (11)	−0.0101 (10)
C141	0.0140 (12)	0.0277 (12)	0.0229 (12)	−0.0086 (10)	0.0037 (10)	−0.0059 (10)
C50	0.0142 (12)	0.0175 (10)	0.0145 (10)	−0.0032 (9)	−0.0027 (9)	−0.0008 (8)
C13	0.0145 (12)	0.0196 (11)	0.0133 (10)	−0.0033 (9)	0.0008 (9)	−0.0025 (8)
C15	0.0118 (12)	0.0185 (11)	0.0199 (11)	−0.0054 (9)	−0.0041 (9)	−0.0016 (9)
C14	0.0114 (12)	0.0131 (10)	0.0210 (11)	−0.0030 (9)	0.0021 (9)	−0.0019 (8)
C642	0.0278 (17)	0.0213 (13)	0.065 (2)	−0.0006 (12)	0.0111 (15)	−0.0078 (13)
C641	0.0290 (15)	0.0182 (11)	0.0273 (13)	−0.0024 (11)	0.0028 (11)	−0.0008 (10)
C33	0.0306 (15)	0.0184 (11)	0.0211 (12)	−0.0052 (11)	0.0008 (11)	−0.0067 (9)
C35	0.0147 (13)	0.0198 (11)	0.0202 (11)	−0.0022 (9)	−0.0024 (9)	−0.0003 (9)
C142	0.0232 (15)	0.0422 (16)	0.0296 (14)	−0.0106 (13)	−0.0002 (12)	0.0071 (12)
C542	0.0304 (16)	0.0261 (13)	0.0292 (13)	−0.0071 (11)	−0.0066 (11)	−0.0086 (10)

### Geometric parameters (Å, °)

**Table:**

Cu1—O3	1.9498 (15)	C31—C32	1.392 (3)
Cu1—O4	1.9501 (16)	C34—C33	1.394 (3)
Cu1—O2	1.9593 (16)	C34—C35	1.397 (3)
Cu1—O1	2.0040 (16)	C34—C641	1.509 (3)
Cu1—O5	2.1761 (15)	C32—C33	1.387 (3)
Cu1—Cu1^i^	2.6047 (5)	C32—H32	0.93
O6—C50	1.326 (3)	C56—H56	0.93
O6—H6	0.82	C12—C13	1.380 (3)
O1—C10	1.277 (3)	C12—H12	0.93
O3—C30	1.267 (3)	C541—C542	1.517 (4)
O4—C30^i^	1.267 (3)	C541—H54A	0.97
O2—C10^i^	1.261 (2)	C541—H54B	0.97
O5—C50	1.223 (3)	C141—C14	1.505 (3)
C51—C52	1.392 (3)	C141—C142	1.532 (3)
C51—C56	1.397 (3)	C141—H14A	0.97
C51—C50	1.479 (3)	C141—H14B	0.97
C11—C16	1.396 (3)	C13—C14	1.400 (3)
C11—C12	1.398 (3)	C13—H13	0.93
C11—C10	1.488 (3)	C15—C14	1.396 (3)
C30—O4^i^	1.267 (3)	C15—H15	0.93
C30—C31	1.501 (3)	C642—C641	1.523 (4)
C53—C52	1.378 (3)	C642—H64A	0.96
C53—C54	1.403 (3)	C642—H64B	0.96
C53—H53	0.93	C642—H64C	0.96
C55—C56	1.386 (3)	C641—H64D	0.97
C55—C54	1.388 (3)	C641—H64E	0.97
C55—H55	0.93	C33—H33	0.93
C52—H52	0.93	C35—H35	0.93
C54—C541	1.505 (3)	C142—H14C	0.96
C36—C35	1.381 (3)	C142—H14D	0.96
C36—C31	1.387 (3)	C142—H14E	0.96
C36—H36	0.93	C542—H54C	0.96
C16—C15	1.382 (3)	C542—H54D	0.96
C16—H16	0.93	C542—H54E	0.96
C10—O2^i^	1.261 (2)		
			
O3—Cu1—O4	169.67 (6)	C55—C56—C51	119.3 (2)
O3—Cu1—O2	89.21 (7)	C55—C56—H56	120.3
O4—Cu1—O2	89.79 (7)	C51—C56—H56	120.3
O3—Cu1—O1	89.64 (7)	C13—C12—C11	120.3 (2)
O4—Cu1—O1	89.46 (7)	C13—C12—H12	119.8
O2—Cu1—O1	169.42 (6)	C11—C12—H12	119.8
O3—Cu1—O5	100.25 (6)	C54—C541—C542	113.8 (2)
O4—Cu1—O5	90.05 (6)	C54—C541—H54A	108.8
O2—Cu1—O5	99.99 (6)	C542—C541—H54A	108.8
O1—Cu1—O5	90.57 (6)	C54—C541—H54B	108.8
O3—Cu1—Cu1^i^	86.32 (5)	C542—C541—H54B	108.8
O4—Cu1—Cu1^i^	83.36 (5)	H54A—C541—H54B	107.7
O2—Cu1—Cu1^i^	87.95 (5)	C14—C141—C142	112.7 (2)
O1—Cu1—Cu1^i^	81.48 (4)	C14—C141—H14A	109.1
O5—Cu1—Cu1^i^	169.69 (5)	C142—C141—H14A	109.1
C50—O6—H6	109.5	C14—C141—H14B	109.1
C10—O1—Cu1	125.79 (14)	C142—C141—H14B	109.1
C30—O3—Cu1	120.62 (14)	H14A—C141—H14B	107.8
C30^i^—O4—Cu1	124.01 (14)	O5—C50—O6	123.3 (2)
C10^i^—O2—Cu1	120.97 (15)	O5—C50—C51	122.7 (2)
C50—O5—Cu1	128.90 (15)	O6—C50—C51	113.97 (19)
C52—C51—C56	119.6 (2)	C12—C13—C14	121.5 (2)
C52—C51—C50	118.4 (2)	C12—C13—H13	119.2
C56—C51—C50	122.0 (2)	C14—C13—H13	119.2
C16—C11—C12	118.5 (2)	C16—C15—C14	120.9 (2)
C16—C11—C10	120.04 (19)	C16—C15—H15	119.5
C12—C11—C10	121.5 (2)	C14—C15—H15	119.5
O3—C30—O4^i^	125.7 (2)	C15—C14—C13	117.8 (2)
O3—C30—C31	117.29 (19)	C15—C14—C141	121.5 (2)
O4^i^—C30—C31	117.03 (19)	C13—C14—C141	120.7 (2)
C52—C53—C54	121.0 (2)	C641—C642—H64A	109.5
C52—C53—H53	119.5	C641—C642—H64B	109.5
C54—C53—H53	119.5	H64A—C642—H64B	109.5
C56—C55—C54	121.9 (2)	C641—C642—H64C	109.5
C56—C55—H55	119.1	H64A—C642—H64C	109.5
C54—C55—H55	119.1	H64B—C642—H64C	109.5
C53—C52—C51	120.3 (2)	C34—C641—C642	110.1 (2)
C53—C52—H52	119.9	C34—C641—H64D	109.6
C51—C52—H52	119.9	C642—C641—H64D	109.6
C55—C54—C53	117.9 (2)	C34—C641—H64E	109.6
C55—C54—C541	121.5 (2)	C642—C641—H64E	109.6
C53—C54—C541	120.6 (2)	H64D—C641—H64E	108.2
C35—C36—C31	120.3 (2)	C32—C33—C34	120.8 (2)
C35—C36—H36	119.8	C32—C33—H33	119.6
C31—C36—H36	119.8	C34—C33—H33	119.6
C15—C16—C11	120.9 (2)	C36—C35—C34	121.1 (2)
C15—C16—H16	119.5	C36—C35—H35	119.5
C11—C16—H16	119.5	C34—C35—H35	119.5
O2^i^—C10—O1	123.7 (2)	C141—C142—H14C	109.5
O2^i^—C10—C11	117.84 (19)	C141—C142—H14D	109.5
O1—C10—C11	118.46 (18)	H14C—C142—H14D	109.5
C36—C31—C32	119.2 (2)	C141—C142—H14E	109.5
C36—C31—C30	120.3 (2)	H14C—C142—H14E	109.5
C32—C31—C30	120.5 (2)	H14D—C142—H14E	109.5
C33—C34—C35	118.2 (2)	C541—C542—H54C	109.5
C33—C34—C641	121.5 (2)	C541—C542—H54D	109.5
C35—C34—C641	120.2 (2)	H54C—C542—H54D	109.5
C33—C32—C31	120.3 (2)	C541—C542—H54E	109.5
C33—C32—H32	119.9	H54C—C542—H54E	109.5
C31—C32—H32	119.9	H54D—C542—H54E	109.5

Symmetry codes: (i) −*x*+1, −*y*+1, −*z*+2.
